# Behavioral, Cognitive and Neural Markers of Asperger Syndrome

**DOI:** 10.18869/nirp.bcn.8.5.349

**Published:** 2017

**Authors:** Farnaz Faridi, Reza Khosrowabadi

**Affiliations:** 1. Institute for Cognitive and Brain Sciences, Shahid Beheshti University, Tehran, Iran.

**Keywords:** Asperger syndrome, Behavioral deficit, Cognitive dysfunction, Neurobiological marker

## Abstract

Asperger syndrome (AS) is a subtype of Autism Spectrum Disorder (ASD) characterized by major problems in social and nonverbal communication, together with limited and repetitive forms of behavior and interests. The linguistic and cognitive development in AS is preserved which help us to differentiate it from other subtypes of ASD. However, significant effects of AS on cognitive abilities and brain functions still need to be researched. Although a clear cut pathology for Asperger has not been identified yet, recent studies have largely focused on brain imaging techniques to investigate AS. In this regard, we carried out a systematic review on behavioral, cognitive, and neural markers (specifically using MRI and fMRI) studies on AS. In this paper, behavior, motor skills and language capabilities of individuals with Asperger are compared to those in healthy controls. In addition, common findings across MRI and fMRI based studies associated with behavior and cognitive disabilities are highlighted.

## 1. Asperger Syndrome

American Psychiatric Association (APA) has recently inserted Asperger syndrome (AS) in the spectrum of Autism Spectrum Disorder (ASD). APA classification has brought many concerns for parents of children with Asperger as they thought this syndrome requires certain caring and training. In addition, experts also think that it is a premature classification because of biological differences between AS and ASD. Therefore in this review, we try to address this concern by looking at behavioral, cognitive and neural differences of patients between Asperger and autism. We would like to show whether these differences are intrinsic or a reflection of developing with different characteristics.

Asperger is defined as a neurobiological disorder that causes obvious deficiencies in social skills such as difficulty in communication and changing the routines (Iwanami et al., 2011). Gillberg defined and characterized Asperger as a social deficiency, limited interest, obligatory behavior with no verbal communication problem (Maier et al., 2002). Although Asperger disorder is considered as high functioning end of autism, there are still some important differences such as normal intelligence and near-normal language development in AS children (Koyama & Kurita 2008, Helles, Gillberg, Gillberg, Billstedt, 2016).

Asperger like autism could not be prevented or treated. However, recent findings indicate that early diagnosis and intervention would drastically improve the treatment process (Lopata, Thomeer Volker, & Nida, 2006) and directly influence the quality of patient’s life. Therefore, to propose a better intervention procedure, it is crucial to look for a subtype diagnosis and consequently a specific intervention. In this regard, we believe that AS must be investigated separately. In this investigation, several co-factors could influence the procedure of diagnosis. Some of the important factors, including effects of age, gender (Asperger individuals and their parents) and Intelligence Quotient (IQ) are reviewed. Also, the effect of each factor on behavioral, chemical and brain structural changes is discussed as follows.

### 1.1. Age effect

One of the main problems in diagnosis of autism and AS is the late occurrence of some atypical behavioral and cognitive changes, like impairment of social communication or alteration in occurrence of puberty. These facts should be considered while proposing a compensatory learning or an intervention method to make it more effective on children or adolescents. In fact, concentration of metabolites, for instance choline, is significantly related to the age (O’Brien et al., 2010). Moreover, metabolites are good indicators of the neural density and their age dependency could also associate well with age dependency of the brain structure. In addition, extent of metabolites is also correlated with behavior and cognitive functions. Therefore, it is necessary to look at metabolic and brain structural changes while investigating the late occurrence of behavioral and cognitive functions. Studies have indicated lower choline concentration in AS individuals with increasing age (O’Brien et al., 2010).

It has also been shown that focalization of all metabolites in prefrontal lobe is higher in subjects with AS (Shamay-Tsoory et al., 2005). There are significant age related differences in terms of pattern of brain structure in Asperger subjects as compared to healthy controls. For instance, volume of cerebral hemispheres and caudate nuclei alter more by the age in AS than healthy control (McAlonan et al., 2002). In addition, maturation of the amygdala and hippocampus complex differs significantly in AS from normal people (O’Brien et al., 2010). For instance, age related increase of amygdala volume (more significant at the left side) is observed in AS people but in normal people (Murphy et al., 2012). These findings also highlight the fact that age is also another important factor for investigation of AS.

### 1.2. Gender effect

Studies commonly report that AS is observed four times more in males than females. However, professionals believe in a prevalence rate of 2:1. In addition to prevalence rate, there are several behavioral and cognitive differences between males and females with AS. For instance, studies have shown that male AS poorly perform the emotion recognition tasks using both face and voice stimuli compared to healthy controls (Helles et al., 2016; Sucksmith, Allison, Baron-Cohen, Chakrabarti, & Hoekstra, 2013). Regardless of diagnosis, female subjects show a better performance in face recognition compared to the males. Furthermore, male subjects in AS group have more difficulty in emotion recognition, and they do worse in the facial emotion recognition than that of voice stimuli (Golan, Baron-Cohen, Hill, 2006). Another behavior study done by Kuhnert shows association of girls prosocial behavior with their emotion understanding but no such association for boys (Kuhnert, Begeer, Fink, & De Rosnay 2017).

Furthermore, girls show more advanced theory of mind compared to the boys in AS (Weimer, Parault Dowds, Fabricius, Schwanenflugel, & Suh, 2017). Gender also affects the neural structure and functions. For instance, it has been reported that males with high-functioning autism and AS have a pattern of decreased grey matter density in the ventromedial regions of the temporal cortex. This fact could influence integration of visual stimuli as well as process of affective information processing (Kwon, Ow, Pedatella, Lotspeich, & Reiss, 2007). Based on the above mentioned studies, biological sex differences should be considered as an important factor in the study of AS and we should not blindly assume that everything found in males applies to females.

### 1.3. Effect of Intelligence Quotient (IQ)

The IQ is mainly measured using two separate components, including performance and verbal levels. Studies have shown verbal and performance IQ differences between AS and normal people, although the findings are controversial (Bölte, Dziobek, & Poustka, 2008; Kanai et al., 2012) The effect of IQ has been more studied in behavioral and cognitive level, therefore, in this section some of the related studies are reviewed.

#### 1.3.1. Performance IQ

A brief report on profiling of the intelligence in AS has been presented by Spek, Scholte, Van Berckelaer-Onnes (2007). The ability was assessed by Wechsler Adult Intelligence Scale (WAIS), which is a valid indicator of IQ (Holdnack, Goldstein, Drozdick, 2011). This test is recommended as the best measurement of IQ in high functioning individuals (Bölte et al., 2008). It is reported that Asperger patients have poor performance in digit symbol coding and symbol search (Kanai et al., 2012). Studies have also supported weakness of AS patients in performance IQ, particularly in digit symbol coding and symbol search (Kuo, Liang, Tseng, & Gau, 2014). Although there is evidence showing deficits of AS patients in coding but they could gain higher score in arithmetic and digit span (Koyama & Kurita, 2008). There are also studies that have focused on reaction time and they believe AS people use significant longer reaction time and need more prompt questions to solve a task (Kaland, et al., 2002). In addition, weakness in processing speed has been reported as well (Holdnack et al., 2011). Therefore, weakness in performance IQ and speed could be good criteria to distinguish AS.

### 1.3.2. Verbal IQ

There is no robust history of language delay or of mental retardation for AS subjects. Typically, AS subjects show above average or superior verbal IQ and high scores on information and vocabulary subtests (Saulnier & Klin, 2006). Studies have shown that AS individuals outperform in the test of fluid reasoning (Hayashi, Kato, Igarashi, & Kashima, 2008). Despite applied use of language, rote linguistic skills such as repeating, appellation, fluency and synaptic comprehension is normal (Saulnier & Klin, 2006; Chevallier, Noveck, Happé, & Wilson, 2009). Therefore, AS subjects have a weak grasp of inference, understand little content, disorganized and narrative, but they show a good vocabulary and grammar skill. In AS individuals, rhythm, volume, and prosody of speech are often disturbed (Chevallier et al., 2009). In other words, AS subjects have more difficulty in brain organization of semantic and syntactic processes. Nevertheless, this deficit has been compensated by other brain regions, allowing AS individuals attain the same level of response activity as controls (Tsai et al., 2013). Therefore, IQ components should also be considered as an important factor while studying AS.

### 1.4. Parents’ gender effect

AS children mostly have similar behavioral traits as their parents. For instance, during cognitive tasks a prolong latency has been reported for AS children as well as for their fathers but not their mothers. Furthermore it has also been reported that the brain responses to sound encoding stimuli are similarly abnormal in both mothers and fathers of children diagnosed with AS. However, during auditory discrimination tasks, only fathers show abnormal patterns (Jansson-Verkasalo et al., 2005). Identical abnormality of dorsolateral prefrontal cortex in both father and son with AS has also been reported (Schultz, Romanski, & Tsatsanis, 2000). An atypical neural response to affective prosody has also been observed in children with AS and their fathers, especially over the right brain hemisphere (Korpilahti et al., 2006). Therefore, parent’s gender could be considered as an important factor while studying AS.

In addition to the concern about covariates in investigating AS, it is important to understand behavioral, cognitive and neural markers of the disorder. Therefore, in this paper we try to review three important aspects of AS, including social behaviors, motor skills and language. We mainly review those studies that have used neuroimaging (MRI, fMRI) from year 1993 onward. In this review, we reported available clinical studies which seem to be relevant to our subject from PubMed, neuropsychology, autism and developmental disorder, biological psychiatry, and journal of physiology. Our search was performed using the following keywords: Asperger, Autism spectrum disorder, high functioning autism, MRI, fMRI, behavior, cognition, and neural marker. Reference list of each article was also assessed for additional citations and those relevant to Asperger studies were also included. It should be mentioned that in this paper only neuroimaging findings based on MRI and fMRI studies were reviewed and other methods such as EEG/MEG as well as non-English studies will be included in our future works.

## 2. Behavioral Markers of Asperger Syndrome

The term behavioral marker refers to a prescribed set of behaviors that could indicate some aspects of a subject’s performance. Performance of a subject could be measured with different techniques. One of the commonly used techniques in this area is eye tracking because of its high reliability. Eye tracking is a process of measuring either points of gaze or motions of the eyes relative to the head (Feng & Cai, 2016). Various theories in psychology could be assessed using this technique. For many decades it has been used for normal population, however, recent research shows a trend toward using this technique on individuals with disorders such as AS (Boraston & Blakemore 2007).

These studies typically focus on the processing of socially salient stimuli and social interactions. So far various eye movement abnormalities, including increased errors and latencies on anti-saccadic task and impairment of pursuit have been reported (Manoach, Lindgren, Barton, 2004), for example, AS individuals mainly focus on mouth region instead of eyes (Sawyer, Williamson, Young, 2011). Moreover, AS individuals do not show the gaze cueing effect. This fact indicates an impairment of unconscious, but not conscious joint attention in AS (Sato, Uono, Okada, & Toichi 2010). In addition, AS people do not show an enhancement of joint attention by fearful vs. neutral gaze which is observed in the control subjects (Uono, Sato, Toichi, 2009). Besides eye-tracking pattern, there are several cognitive abilities affected in AS. Some of the important impaired abilities are explained in the following.

AS is also characterized by stereotypic and obsessional behavior and abnormalities in socioemotional and communicative behavior (McAlonan et al., 2002). Some AS patients are known to commit offences and more likely violent offences against strangers (Tantam & Girgis, 2008). Moreover, a lower score on the Friendship Questionnaire (FQ) has been reported in AS subjects (Baron-Cohen & Wheelwright, 2003). Lack of sense of humor has also been reported, although anecdotal and prenatal reports provide some evidence to the contrary (Lyons & Fitzgerald 2004). Furthermore, inability to laugh at themselves (gelotophilia), but enjoy laughing at others (katagelasticism) has also been reported in AS group (Kaplar, 2011). According to Helles findings, there is a characteristic discrepancy between AS pure (no current psychiatry comorbidity) and AS plus (AS and one other current disorder). AS pure shows indifferent and detached behavior while AS plus are worrying, fearful, shy and fatigable (Helles et al., 2016).

### 2.1. Irregularity of movement in Asperger syndrome

Motor abnormality includes variety of impairments; inability to execute a sequence of actions, atypical eye movement and deficiencies in motor learning (Setoh, Marschik, Einspieler, & Esposito, 2017). There are several studies examining movement in AS patients, presenting abnormalities in motion performance (Price, Edgell, Kerns, 2012). In addition, different visual sensitivity to the movement and postural responsivity to the optic flow, correlate with motor skills (Price, Shiffrar, Kerns, 2012). There is also significant impairment of movement performance as well as proprioceptive and vestibular processing in AS (Siaperas et al., 2011). Furthermore, AS individuals show deficit in explicit motor-sequence learning that could be related to the left hemisphere dysfunction (Izadi-Najafabadi, Nejati, Mirzakhany-Araghi, & Pashazadeh-Azari, 2013). Explicit learning of right and left hand movement is impaired in AS. However, despite the impairment of explicit learning (Watanabe, Ikeda, Miyao, 2010), the implicit learning of both hands maintains intact. Attractively, a right hand preference in implicit motor learning is observed in AS individuals due to left striatal system abnormality (Izadi-Najafabadi et al., 2013).

## 3. Cognitive Markers of Asperger Syndrome

AS adults have distinct pattern of cognition from healthy control (Bucaille et al., 2016). Several areas of the cognitive style and functions are disrupted in AS such as social skills (Myles & Simpson, 2001), joint attention (Tanguay, Robertson Derrick, 1998), central coherence (Jolliffe & Baron-Cohen, 1999), executive functioning (Semrud-Clikeman, Walkowiak, Wilkinson, & Butcher, 2010) and implicit social cognition (Lugnegard, Unenge Hallerbäck, Hjärthag, & Gillberg, 2013). There are several tasks, which could be used to measure cognitive abilities in AS subjects such as Wechsler intelligence scale, Neuropsychological Assessment (NEPSY), reading the mind in the eyes, Multifaceted Empathy Test (MET), folk psychology, folk physic test, reading the mind in the voice, and embedded figure test. Therefore, some of the related studies are reviewed in the following.

It has been reported that AS patients show decreased pointing accuracy, timing accuracy and postural stability (Gowen & Miall, 2005). They recall fewer memories, which are often rated as known. They show fewer social identities and more abstract and trait-linked identities (Tanweer, Rathbone, Souchay, 2010). In addition, AS individuals have significant impairments on test of visual memory and on executive functioning, flexibility and generativity (Ambery, Russell, Perry, Morris, & Murphy, 2006). In addition, AS individuals experience difficulties with extra-dimensional/conceptual shifts (between categories or rules) (Brady et al., 2013). Interestingly, they do not differ from controls in their judgments of causality or blame judgment in relation with non-mentalistic factors. In contrast, they are more sensitive to mentalistic considerations in their attributions of blame. They make greater differentiation between intentional and unintentional actions as compare to controls. They also better differentiate between actions with likely protagonists believed versus unlikely ones that lead to negative consequences (Channon, Lagnado Fitzpatrick, Drury, & Taylor, 2011).

In general, AS children respond faster and more accurately to expression of happy faces than controls (Wong, Beidel Sarver, & Sims, 2012). However AS and control do not differ in their emotional expression and responses to facial stimuli (Doody & Bull, 2012). Moreover, AS individuals perform as accurate as controls at matching fear body postures, but less accurate than controls in verbally identifying the same stimuli (Doody & Bull, 2012). Moreover, individuals with AS may have an impaired response to change their environment (Brosnan, Turner-Cobb, Munro-Naan, & Jessop, 2009). They also fail to show interaction effect (Shamay-Tsoory, Gev, Aharon-Peretz, & Adler, 2010). The AS subjects are impaired in folk psychology but superior in folk physics (Baron-Cohen, Wheelwright, Spong, Scahill, & Lawson, 2001). Another key feature of AS is lack of theory of mind (Nieminen-Von et al., 2003, Frith, 2004). Moreover, the cognitive planning is also weaker in AS individuals (Ashley Jones Reno, 2013). However, they outperform on the fluid reasoning test (Hayashi et al., 2008). It is also interesting that AS subjects are impaired in cognitive empathy, but they do not differ from controls in emotional empathy (Dziobek et al., 2007). Similarly, O’Brien reported the intactness of affective empathy and recognition of negative emotions in AS (O’Brien et al., 2010).

### 3. 1. Language domain in Asperger syndrome

There is no difference between High Functioning Autism (HFA) and AS in verbal skills but their history of language acquisition is different (Bennett et al., 2007). History of language acquisition and the time at which language is acquired, is crucial for configuration of AS phenotype (McAlonan et al., 2009). AS individuals show no problem in literal semantic processing, which is a rule-based task. However, they have deficit in violating processing, like metaphors (Gold & Faust, 2012). They make more mistakes in appropriate metaphoric sentences than in scrambled metaphors. These findings indicate intact automatic metaphor processing in AS (Hermann et al., 2013). In addition to metaphor comprehension (Gold, Faust, Goldstein, 2010), AS individuals have difficulty in social interactions and understanding sarcasm (Smucker, 2011) which are related to less activity of right brain hemisphere in AS group.

In contrast, some studies attribute difficulties in the comprehension of metaphors in AS to differences in linguistic information processing (Gold et al., 2010). Problems in audio speech perception has been reported (Saalasti et al., 2011). Similar findings have been observed in visual articulation as well. This may relate well with the difficulties in face to face communication in AS individuals (Saalasti, Tiippana, Kätsyri, & Sams, 2011). Moreover, spreading grammatical prosody has also been indicated in AS (Chevallier et al., 2009). It is interesting that AS individuals are unable to talk about their own emotions but they can write about their lives (Frith, 2004). Fortunately, despite the changes in organization of the brain for semantic and syntactic processing in AS group, the other brain mechanisms (top-down regulation) can compensate the deficits and allow AS to attain the same level of response and activation as healthy controls (Tsai et al., 2013).

In other words, the above mentioned behavioral and cognitive dysfunctions have neurobiological basis that requires to be reviewed as well. Since the brain is the center of cognitive functions, a comprehensive review requires covering findings on structural and functional changes of the brain in AS subjects. Anatomical structure of the brain is normally investigated by Magnetic Resonance Imaging (MRI) (Berthier, Bayes, Tolosa, 1993). MRI technology could also be used to measure brain functions by tracking the hemodynamic response (BOLD signal) in response to stimuli. Therefore, this technique has largely been used to track anatomical and functional changes of the brain. The following section provides a review on recent findings on structural and functional differences in AS individuals.

## 4. Neurobiological Basis of Asperger Syndrome

AS causes some chemical, structural and functional abnormalities in the brain which are discussed in following.

### 4. 1. Chemical markers

There are several neurotransmitters responsible for dampening or facilitating cellular activities in the brain. Studies have shown that concentration of neurotransmitters are different in AS and it influences the brain functioning pattern. A higher level of N-acetyl aspartate/choline (NAA/Cho) level in AS has been found at the right anterior cingulate (Oner, Devrimci-Ozguven et al. 2007). In addition, [^18^F] F-Dopa influx(k) increase in the striatum, putamen, caudate nucleus and frontal cortex has been reported (Nieminen-von Wendt et al., 2004). This clearly indicates that dopaminergic system is largely affected in AS.

It should be mentioned that association between changes in neurotransmitter levels and cognitive behavior has also been explored in AS. For instance, changes in level of NAA/Cho are positively correlated with the obsessive compulsive scale that is affected in AS. Moreover, changes in modulation of serotonin in the brain by acute tryptophan depletion could lead to deviation in the processing of emotional faces in male AS compared to controls (Daly et al., 2008). In addition, intranasal administration of the neuropeptide oxytocin could improve performance of the AS subjects in a facial emotion recognition task (Domes, Kumbier, Heinrichs, & Herpertz 2013). Similarly, Domes group suggested that applying oxytocin may improve affective speech comprehension and increase eye gaze, emotion recognition as well as social interaction (Domes et al., 2013). Beside the neurochemical differences in AS individuals, there are some brain structural changes that are listed in the following.

### 4. 2. Brain structural changes

The spread and heterogeneity of the neuroimaging findings about AS suggest that it is a widely distributed disorder affecting both grey and white matters. Volume of grey and white matter of some brain regions are different in AS. Neuroimaging studies have indicated lower grey matter volumes in the bilateral amygdala, hippocampus gyrus, prefrontal lobe, medial frontal gyrus, left occipital gyrus, right cerebellum, limbic striatal, bilateral caudate, left thalamus, putamen and precuneus as compared to healthy controls (McAlonan et al., 2008; Ameis et al., 2011, Semrud-Clikeman & Fine, 2011).

In addition, greater grey matter volumes have also been observed in AS at the bilateral inferior parietal lobule and the left fusiform gyrus. Moreover, a higher volume of white matter has been reported around the basal ganglia, left parietal lobe, but a lower white matter volume has also been observed at the right frontal region and corpus callosum (McAlonan et al., 2009). In addition to abnormalities in white and grey matter, the anterior posterior diameters of the mesencephalon are significantly shorter in AS (Nieminen-von Wendt et al., 2002).

In terms of structural organization of the brain, AS patients have lower fractional anisotropy in the short intracerebellar fibers and right superior cerebellar peduncle. This has been reported to be largely bilateral and includes white matter in the internal capsule, frontal, temporal, parietal and occipital lobe, cingulum and corpus callosum (Bloemen et al., 2010). Furthermore, abnormality in volumes of hippocampus, amygdala and Anterior Cingulate Cortex (ACC) has been reported. The brain anatomical and structural abnormalities cause the cognitive deficits in AS. For instance, the abnormalities at the ACC, amygdala and hippocampal regions in AS are likely the main contributor of their difficulty with modulating of emotional reactivity (Semrud-Clikeman, Fine, Bledsoe, & Zhu, 2013). Moreover, the localized cerebral abnormalities in AS discharge adaptive social behavior (Catani et al., 2008). In addition, volumetric exes in the Asperger group at the inferior parietal lobule is linked to synesthesia (Welchew et al., 2005) and dysfunction within frontostriatal and cerebellar motor circuits also reflect chaotic movement (Nayate, Bradshaw, Rinehart, 2005). Furthermore, association between abnormalities and size of some particles in the brain has been observed. For instance, measures of entropy and uniformity have been reported to be related to the volume of the caudate nuclei. In terms of global features, average of the grey matter volume is related to the size of the cerebellar vermis (Radulescu et al., 2012) and there is no positive relationship between the total brain volume and size of thalamus in AS group (Hardan et al., 2007). In addition to brain structural differences in AS individuals compared to healthy controls, lesions could also be found mainly in occipital lobe in AS group, on the areas responsible for visual/spatial reasoning (Semrud-Clikeman & Fine, 2011).

### 4. 3. Brain functional changes

Functional studies with the help of pattern recognition techniques are converging on the hypothesis that AS is associated with atypical decreased functional connectivity between nodes in the default mode network (Funai, Bharadwaj, Grissom, 2007) and executive control network (Han & Chan, 2017). Functional abnormalities exist in cerebella, frontal and temporal lobes, and limbic system (Sugihara, Ouchi. Nakamura, Sekine, & Mori 2007). In addition, significant abnormality in the functional integration of amygdala and parahippocampal gyrus have also been observed (Welchew et al., 2005). Interestingly, neuroimaging patterns of AS individuals are not affected by stimuli type (static and dynamic faces) (Horlin et al., 2013). For instance, story task and cartoon task activate the same region of brain (medial prefrontal region) (Castelli, 2002). Additionally, main emotions, manifested by face, can activate fusiform and extra striates (Deeley et al., 2007).

Despite the believe that neurobiological underpinning in AS and High Functioning Autism (HFA) are not separated, recent studies indicate that orbitofrontal functionality compromises in HFA but integrates in AS (May et al., 2010). However, similarities are also observed in both groups for instance in striate and extra-striate areas (Bókkon, Salari, Scholkmann, Dai, & Grass, 2013). After all, the most dominant aspects of diagnosis of AS are behavior, then language and movement. Therefore, in the following sections, we will review articles published on the above mentioned domains in AS.

## 5. Comorbidity of Asperger Syndrome and Psychiatric Disorders

Many AS individuals develop secondary psychiatric disorders in adolescence and adulthood. This could be because of genetic, psychiatric family history, and neurological diseases in autistic spectrum disorders. The most common comorbidity include Attention Deficit and Hyperactivity Disorder (ADHD), depression, or both (Gillberg, Helles, Billstedt, & Gillberg, 2015). They more likely to have relatives with depression, schizophrenia, and broader autistic phenotype. Moreover, AS seems to be more prevalent in adults with ADHD. Along with above mentioned disease, bipolar disorder in AS is frequently observed (Vannucchi et al., 2014).

Studies have also shown that persons with frontotemporal lobe degeneration, like those with maladaptive behavior, may suffer from subclinical AS, too. Gender dysphoria (Shumer, Reisner, Edwards-Leeper, & Tishelman, 2016) and NLD (Hagberg, Billstedt, Nydén, & Gillberg, 2015) as well as Post-Traumatic Stress Disorder (PTSD) (Ipci, Inci, Akyol Ardiç, & Ercan, 2017) have also been reported to be comorbid with AS. These studies report coincidence of Asperger and other disorders. Therefore, investigation of these comorbidities and brain structural similarities among AS people and individuals with ADHD, schizophrenia, depression and anxiety are also recommended for future studies.

In summary, several findings on abnormalities of behavior, cognition and brain structure and functions in AS were discussed. According to previous studies, the most apparent characteristics of AS individuals are the stereotypic and obsessive behavior as well as abnormalities in socioemotional and communicative behavior. Furthermore, they show more irregularities in motor development. Moreover, history of language acquisition is also important. In other words, better understanding of behavioral and cognitive abnormalities requires a better knowledge of neural alteration in AS. In general, studies point to structural abnormalities in AS such as decrease in grey matter at temporal cortex, bilateral amygdala, hippocampus gyrus, prefrontal lobe, medial frontal gyrus, left occipital gyrus, right cerebellum, limbic striatal, bilateral caudate, left thalamus, putamen and precuneus. There are also functional abnormalities in cerebellum, frontal, temporal lobes, and limbic system.

In addition, male AS are considerably more than females and since functional abnormalities of AS children are similar to their fathers, it is also advisable to look at parent/gender effect as well. The findings could help on early diagnosis and proposing an earlier intervention program.
